# Detection of Ponzi scheme on Ethereum using machine learning algorithms

**DOI:** 10.1038/s41598-023-45275-0

**Published:** 2023-10-27

**Authors:** Ifeyinwa Jacinta Onu, Abiodun Esther Omolara, Moatsum Alawida, Oludare Isaac Abiodun, Abdulatif Alabdultif

**Affiliations:** 1 Department of Computer Science, University of Abuja, Gwagwalada, Nigeria; 2 Department of Computer Sciences, Abu Dhabi University, 59911 Abu Dhabi, United Arab Emirates; 3 Department of Computer Science, College of Computer, Qassim University, Buraydah 52571, Saudi Arabia

**Keywords:** Engineering, Mathematics and computing

## Abstract

Security threats posed by Ponzi schemes present a considerably higher risk compared to many other online crimes. These fraudulent online businesses, including Ponzi schemes, have witnessed rapid growth and emerged as major threats in societies like Nigeria, particularly due to the high poverty rate. Many individuals have fallen victim to these scams, resulting in significant financial losses. Despite efforts to detect Ponzi schemes using various methods, including machine learning (ML), current techniques still face challenges, such as deficient datasets, reliance on transaction records, and limited accuracy. To address the negative impact of Ponzi schemes, this paper proposes a novel approach focusing on detecting Ponzi schemes on Ethereum using ML algorithms like random forest (RF), neural network (NN), and K-nearest neighbor (KNN). Over 20,000 datasets related to Ethereum transaction networks were gathered from Kaggle and preprocessed for training the ML models. After evaluating and comparing the three models, RF demonstrated the best performance with an accuracy of 0.94, a class-score of 0.8833, and an overall-score of 0.96667. Comparative evaluations with previous models indicate that our model achieves high accuracy. Moreover, this innovative work successfully detects key fraud features within the Ponzi scheme dataset, reducing the number of features from 70 to only 10 while maintaining a high level of accuracy. The main strength of this proposed method lies in its ability to detect clever Ponzi schemes from their inception, offering valuable insights to combat these financial threats effectively.

## Introduction

The conviction of Bernard Madoff, an American investment adviser, and his 150-year prison sentence in 2009 for orchestrating a $65 billion Ponzi scheme, raised public concern about this form of online financial fraud^1,2^. It has also enticed some individuals to seek quick ways of making money^3,4^ while increasing awareness of investment and securities fraud in the financial industry^2^. With the introduction of Ethereum to blockchain technology between 2014 and 2015, scammers found an ideal platform due to its decentralized nature, making it popular among Ponzi scheme operators. Ethereum, an open-source system running on blockchain technology, supports smart contracts and decentralized applications, providing researchers access to transaction records for scam detection. Ponzi schemes promise high returns to investors within a short period, where new participants fund the returns for earlier investors. When new participants stop joining, the scheme collapses, and those at the top receive their investments, while those at the bottom lose theirs. Scheme initiators consistently assure participants that their funds will be invested wisely to yield substantial returns swiftly.

However, the reality is quite different. Instead of investing the generated funds into viable businesses, the initiators use the funds from later participants to pay off earlier participants. In this process, they divert larger amounts into their own accounts^5^. Ponzi schemes were named after Charles Ponzi in 1919. He convinced investors that they could earn a 50% return on their investment within a few months. These schemes have persisted for over 150 years and have adapted to use cryptocurrencies like Ethereum for currency exchange^6^. According to the Nigerian Security and Exchange Commission (SEC) report (2022), Ponzi scheme promoters have defrauded Nigerians of over N147 billion from 2019 to 2020. The consequences have been severe, with some victims experiencing heartbreak and even resorting to suicide. It is imperative for the government to urgently investigate and apprehend those behind these Ponzi schemes and bring them to justice. Oji (2021) suggests that increasing public awareness about the risks associated with investing in such schemes can help mitigate their impact.

**Figure 1:**
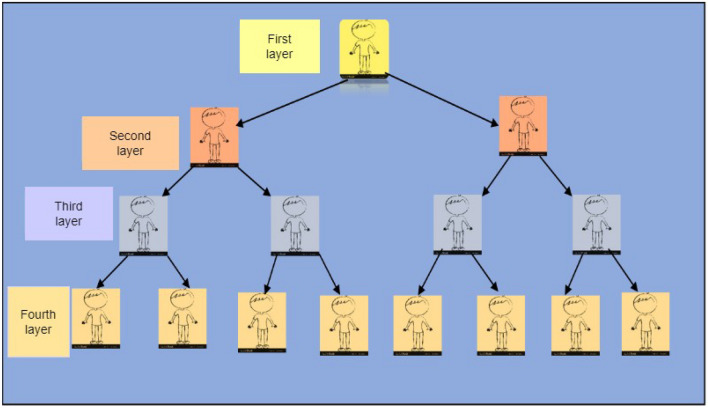
Ponzi scheme contract categorization.

In the Ponzi scheme contract category, the first layer allocates funds to investors based on a predetermined set of rules or principles. But in the second layer, the contract solely acquires funds from investors. However, in the third stratum, each investor derives a profit if a sufficient number of investors allocate a substantial amount of capital to the contractual agreement subsequently. The risk of investment loss increases as an investor joins the contract at a later stage in the fourth layer. Therefore, the payout tree for a program that takes exactly 1 ETH from each user and doubles their investment is shown in Fig. 1

Figure 1 is a representation of how Ponzi schemes work to deceive target people. The owner receives the first ether, that is, a Ponzi scheme is a fraudulent investment scheme that entices investors with high rates of return and little risk. A Ponzi scheme is a fraudulent investment operation in which money is collected from later participants to pay returns to earlier investors. This is comparable to a pyramid scam in that both rely on new investors’ money being used to reimburse the previous funders. When the influx of new investors stops and there isn’t enough money to go around, both Ponzi schemes and pyramid schemes finally hit their bottom. The plans then start to fall apart. According to skeptics such as Roubini and Quinn, cryptocurrencies such as Bitcoin operate similarly to Ponzi schemes, with new investors paying out early investors because no genuine currency flows are produced. Ponzi and pyramid schemes involve unethical investors defrauding naïve people by promising them enormous profits in exchange for their money.

Investors in Ponzi schemes donate money to a portfolio manager and are paid out with monies contributed by later investors. Based on crypto-analysis report on virtual currency scams (investors.org, 2022), over 750 million Naira has been lost to Ponzi schemes. Based on their report there is an indication that financial security^7^ is a serious issue in the blockchain ecosystem^8^. Hence, identifying Ponzi schemes in Ethereum will protect investors’ interests and at the same time reduce the losses incurred by investors. This will equally enable the agencies responsible for the supervision of the Ponzi schemes to supervise the fraudulent activities of fraudsters. Experts^5^ have reported that the damages caused by Ponzi schemes call for the attention of the regulatory bodies to shut them down at an early stage before they gain ground. Hence, stressing the need to inform the public on time and publicize lists of individuals or organizations involved in such illegal Schemes.

This is in line with Abiodun et al.^9^ who stated that security often serves as a critical reason for the widespread adoption of any scientific innovation. Therefore, one of the solutions is to detect Ponzi schemes in time to pass warning information to investors. Therefore, from various reports, it is very clear that Ponzi schemes have caused a lot of damage to investors^10,11^. There is a need to derive a means to combat its negative effect on Nigeria and the global society at large. Therefore, this paper presents a Ponzi scheme detection model that is efficient, explainable, and highly accurate using machine learning algorithms and feature engineering. Machine learning techniques such as RF, KNN, and NN were used in this study because of their previous ability to address the detection of fraud in a network system.

### Paper organization

The whole paper was divided into the following sections: The introduction, which includes the study’s past events, the paper’s structure, and its main contribution, is presented in “Introduction” section. “Smart Ponzi schemes and identification” section explains how to recognize Smart Ponzi Schemes. The associated works are discussed in “Related works” section. In the meanwhile, “Methodology” section explained the procedure for identifying Ponzi schemes. Additionally, “Experimental result” section included the experiment’s findings. Furthermore, “Discussion of the result” section covered the findings, and “Conclusion” section concluded by outlining the results, making recommendations, and suggesting further research.

### Key contributions

The work’s contributions are as follows:

1. Enhancement of Ponzi scheme detection on Ethereum through novel ML algorithms like RF, NN, and KNN.
2. Early detection of Ponzi schemes to prevent financial damage and protect potential victims.
3. Experimental results show the detection and mitigation of security threats posed by Ponzi schemes, which carry higher risk compared to many online crimes.
4. Based on the experimental findings, we recommend developing a standard platform to assess and keep track of each smart contract that is formed to spot fraud in its earliest stages.

## Smart Ponzi schemes and identification

Decentralized applications (DApps), operating on an immutable blockchain, offer users a sense of security. Unfortunately, criminals exploit this feature to create a new type of scam application known as Smart Ponzi Schemes. These apps lack inherent value and rely on constantly attracting new users to pay fees and generate income for their operators. While early participants may benefit, the majority will suffer significant losses due to this wealth redistribution mechanism, hindering the growth of the blockchain ecosystem^12,13^.

Ponzi schemes are a common type of scam that relies on new investors’ investments to pay off the previous ones. The scammers attract new investors by promising high profits and continue to operate as long as they can lure in new funds. However, Ponzi schemes eventually collapse due to the difficulty of sustaining a continuous flow of new investors. With the emergence of blockchain and smart contracts, a new form of smart Ponzi schemes has emerged. These schemes use cryptocurrencies as revenue and take advantage of the immutability and anonymity of the blockchain to make investors’ losses irrecoverable. The combination of new technologies and the allure of high profits exposes a large number of regular investors to fraud.

Take for instance, the Ponzi Scheme game Fomo3D1 quickly overtook Cryptokitties2 to rank among the top games available on the Ethereum platform. According to reports, the game’s first-round winner received a payout of more than $3 million, while most regular players lost their initial investment^14^. Smart Ponzi schemes will cause a lot of significant people to lose money and bitcoin, which is not good for the ecology of blockchain technology as it develops. Smart Ponzi scheme detection is thus a crucial step in maintaining the DApp ecosystem, just as it is crucial to identify harmful apps in Android stores^15^. Keep in mind that the concept of a "DApp" is more user-focused. A DApp is typically made up of a web client for user interaction and some basic smart contracts to run the application. Since smart contracts serve as the foundation for DApps, the term "smart Ponzi scheme" refers to specific smart contracts that implement the Ponzi scheme theory. Therefore, it is implied that only this particular portion of the DApp is using the Ponzi scheme when Ponzi logic is found in a specific smart contract. If and only if a participant’s transactions involve this address, the identification mechanism can send them a warning message. Doing this can safeguard users from participating in Ponzi schemes while limiting the impact on the DApp’s other beneficial features.

While it is possible that many of the participants in the prior example Fomo3D were aware of the risks, there are numerous other smart Ponzi schemes that entice unsuspecting investors under the pretense of an investment plan with high returns^16^. Additionally, some smart Ponzi schemes (i.e., hidden smart Ponzi schemes do not provide source code^17^, in which case even experts are unable to determine whether the scheme is a smart Ponzi scheme. It is vital to research the identification method of a smart Ponzi scheme given the rapid development of blockchain technology, the bulk of users involved lack of professional understanding, and the relatively lax supervision. Therefore, it is very urgent to study the identification method to provide an early warning for people who might fall victim to being defrauded of their money.

## Related works

Bernie Madoff perpetrated the largest Ponzi scam, defrauding thousands of investors of billions of dollars^4,18,19^. The scam’s foundation of the Ponzi scheme is deceit and lies, and it relies on both to deceive innocent investors by encouraging their conviction that they will receive the anticipated rewards^20^. The operator of the Ponzi scheme makes use of the Ethereum platform to achieve their goal^16,21,22^. Ethereum is a decentralized blockchain platform that creates a peer-to-peer network for securely executing and verifying application code known as smart contracts. Smart contracts enable participants to transact with one another without the need for a trusted central authority. Nowadays, the speedy growth of blockchain has brought about increasing users to be attracted to the blockchain ecosystem and many implementations have been carried out in different fields, especially in the crypto-currency investment field. Blockchain technology in recent times has shown robust activeness. However, along with the rise in online business, uncountable fraudulent activities, e.g., money laundering, bribery, Ponzi schemes, and others, have been seen as the main threat to online business security^23^. Hu et al.^24^ and Wang et al.^25^, stated that the high rate of communication on social media has prompted many fraudsters to take advantage and send their fraudulent links on social media in order to scam its users.

A Ponzi scheme is an investment scam that involves the payment of unrealistic returns to its early investors from funds invested by new investors (SEC, 2018)^26^. It is a fraudulent investment activity where the initiator generates returns for older investors through revenue paid by new investors, rather than from legitimate businesses. Ponzi schemes hurt the economy of any country involved and are unacceptable in many countries including Nigeria. This is because in Ponzi schemes, later participants will lose most of their invested money^27^. He further emphasized that all Ponzi schemes are making large amounts of money off people who want to participate in blockchain technology but do not understand how it operates.

Bartoletti et al.^5^ described Ponzi schemes as financial frauds under the promise of high profits. They stressed that the Federal Government of Nigeria has made an official release, intimating citizens about its non-readiness to support the use of Ethereum and at the same time warning individuals, corporate bodies, and banks not to get involved in Ethereum transactions in which most of the Ponzi schemes operate on. This warning was because of the devastating effects of the various Ponzi schemes in Nigeria which collapsed towards the end of the year 2016 up to the first quarter of 2017 when many Nigerians lost their wealth. The Federal Bureau of Investigation (FBI) warns investors to be careful about investment proposals that promise to pay huge amounts of money in a short period of time, which are not backed up by any documentation or quarterly/annual reports^28^. They equally advised the general public to be careful not to invest based on what they will gain alone, but also consider the loss that might align with it, and not to trust investment offerings posted on social networking sites and chat rooms. They also recommended that investors seek third-party advice, for example, to contact an independent broker or licensed financial adviser before investing.

Research has shown that some of the common features of fraudulent schemes are mouth-watering returns and no negative impacts^1,29^. Findings suggest that increased regulatory supervision with tight requirements for compulsory reporting of their activities will help to identify such schemes^30^. Obamuyi et al.^31^, emphasized that after the demise of the Mavrodi Mundal Movement (MMM), which crashed in the year 2016/2017 with a total investment loss of 12 billion nairas on participants. She emphasized bitterly the rising cases of Ponzi schemes that are recently experienced by the citizens such as Racksterli introduced by Michael Chidiebere Oti after the Covid-19 pandemic in the year 2020. The platform offers a monthly profit called Racksterli package to its participants.

Many Nigerians fall victim to this scam because they are looking for such an opportunity to make fast money out of hardship after the economic setbacks experienced during the COVID-19 pandemic. According to her findings, the platform claimed that the invested funds would be used to reinvest in businesses like real estate businesses, forex trading, and cryptocurrency businesses, promising them that the realized funds from such businesses would be used to reward them. But the scheme finally collapsed when there were no more new investors, making old investors lose their invested funds. This is an indication that the initiator did not invest the generated funds in any business as promised. But the question is “Why is Oti Chidiebere the founder of Rocksterli not persecuted”. Perhaps the Oti Chidiebere case is still under investigation and will soon face criminal justice. Another such case is that of the 86fb Ponzi scheme which collapsed in early May 2022. The platform since May 2022 has been having issues paying its participants, deceiving their participants that the problem is from the flutter wave payment gateway. But all these crises experienced in these Ponzi schemes should be a reminder to people who are investing in such unverified Ponzi schemes that promise high returns to investors is very dangerous. Another question is despite the loss and warnings of these Ponzi schemes, why do people still fall victim? The answer is; that Nigerians have shown that they long for high-risk and high-return investments, despite their mode of operation^32^. Supporting the above answer, Chiluwa et al.^1^ stated that it is due to a low amount of low-risk savings promised by the initiator of these schemes to its participants to go against inflation. Oyedeji et al.^33^ stated that many people fall victim to these Schemes because of the following reasons:

1. They prefer investing their money in any investment that will double their invested money within a short period of time than working for it.
2. Due to the problem of poor education and lack of awareness.
3. In order to save long-term wealth in real terms in the financial system.

According to him, these Schemes will continue to scam people because of the inability of the monetary policy to deliver real risk-free-return investments, making the risk-return investments strive deeply in society. There is a need for better investor education and an effective detection model for the Security and Exchange Commission to do a better job of monitoring Ponzi schemes^1^. In line with the above statements, the SEC introduced the whistleblower bounty program.

The program promised that any citizen who reports fraud to the SEC can get up to 30 percent of the penalties and funds recovered from the fraudster. Again, the law also establishes a single toll-free number as a consumer hotline to report any fraudulent act, such as a Ponzi scheme. It suggests that hedge funds and private equity advisers should register with the SEC, in other to establish greater supervision^34^. In fact, the rising rate of Ponzi scheme scams is due to the weak attitude of the regulatory body towards identifying and persecuting their perpetrators^35^. But there is hope as the Ponzi schemes keep increasing; it enforces the Security and Exchange Commission (SEC) to press them down. The SEC has announced its readiness to work with the National Orientation Agency (NOA) and other government agencies to stop the operations of Ponzi schemes and other illegal investment platforms in society (SEC, 2022). Unfortunately, just like other crimes such as terrorism and security challenges^36^, the Ponzi scheme is a rapidly growing crime in society.

More work achieved on Ponzi scheme detection includes the recent work by Zhang et al.^37^ utilized machine learning techniques to address the issue of model stealing attacks on a network. Liu et al.^38^ introduce a unique video self-supervised learning framework called Temporal Contrastive Graph Learning (TCGL), which uses a hybrid graph contrastive learning technique to model both the inter- and intra-snippet temporal dependencies for learning temporal representations. Liu team’s experimental findings show that TCGL outperforms cutting-edge techniques on large-scale action recognition and video retrieval benchmarks in a network. Zhang and Zhou proposed in 2022 that feature pyramid networks (FPN), an essential component of general-purpose object detectors, can greatly improve detection performance for objects of various scales. Bayesian learning is used by Xie et al.^39^, and Cheng et al.^40^ in a separate work to permit the evaluation of uncertainty with regard to the made predictions. Due to their exceptional ability to marginalize uncertainty associated with the parameter estimates, they can more effectively utilize relevant prior information and organically add robustness to the model. Furthermore, the training data can be used to learn the hyper-parameters related to the adopted priors.

A dynamic event-triggered security control issue for networked control systems vulnerable to deception attacks was also the subject of certain investigations. To reflect randomly occurring cyber-attacks, Li et al.’s new work^41^ uses two sets of separate stochastic sequences. The paper by Li et al.^42^, looked at the H consensus issue for multiagent supply chain systems with changeable topology and ambiguous demand. Zhu et al.^43^, offer a simple and effective Siamese-oriented Region Proposal Network (Siamese-ORPN) for visual tracking in their paper. The diversity of visual-semantic hierarchies can yield significant benefits in feature extraction and feature fusion. Personal gadgets, which are backed by a variety of smartphone apps, allow consumers to access Internet services in a convenient and ubiquitous manner, according to Li et al.^41^. The survey summarizes modern technology as well as critical patterns in smartphone app usage behaviors, all of which have major implications for every relevant stakeholder. Li and Sun^44^ investigated the subject of stock intelligent investment strategies based on the support vector machine parameter optimization technique.

In the stock market forecast, the evolutionary algorithm parameter optimization under the radial basis kernel function exhibits the best prediction effect, which is the closest to the true value. Cao et al.^45^ investigated the topic of stock intelligent investing strategy based on the support vector machine parameter optimization technique. The experimental findings suggest that the new method can effectively optimize four model indicators. Yan et al.^46^ present a new technique to enable the realization of the effect of social co-governance based on the innovative properties of the blockchain such as non-tampering, consensus mechanism, and a smart contract. The consensus outcomes created by credible data uploading, model development, and data derivation via smart contracts can considerably reduce the problem of government administrative oversight involution; and the potential for social co-governance. Blockchain smart contracts have given rise to a slew of intriguing and appealing applications, and they have emerged as a transformative force for the Internet. To ensure network vulnerabilities are detected, the research by Liu et al.^47^ focuses on Rethinking Smart Contract Fuzzing: Fuzzing with Invocation Ordering and Important Branch Revisiting. In addition to applying blockchain technology to the health care service system, the paper by Li et al.^48^ concentrates on network vulnerability detection via Efficient Medical Big Data Management with Keyword-Searchable Encryption in the Health chain.

Similarly, Wang et al.^49^ focus on Blockchain-Empowered Distributed Multi-Camera Multi-Target Tracking in Edge Computing to ensure the detection of network vulnerabilities. Due to the intricacy and high dynamics of cloud environments, it is difficult to detect anomalies caused by irregular data fluctuations and to create robust models. To address these concerns, Song et al.^50^, published a paper to identify performance deviations in volatile cloud environments: A robust, explainable, correlative graph neural network-based method for detecting network vulnerabilities. Likewise, a paper by Zenggang et al.^51^ employs the Social Similarity Routing Algorithm based on Socially Aware Networks in the Big Data Environment to detect network vulnerabilities. Moreover, Han et al.^52^ use Practical and Robust Federated Learning With Highly Scalable Regression Training to detect network vulnerabilities.

In addition, Ni et al.^53^ examined the community partition issue under the IC model in social networks. The goal is to maximize the total influence propagation of a social network by optimizing it within each community in order to detect network vulnerabilities. Li and Liu’s^54^ focus was on Scheduling uniform machines with restricted assignments in order to detect network vulnerabilities. Again, Li^55^, proposed efficient algorithms for scheduling tasks of equal length with processing set restrictions on uniform parallel batch machines. Then, Li study’s results extend previous findings for identical machinery. The work of Liu et al.^56^, introduces Visual Question Answering (VQA), which is an important cross-disciplinary issue in the fields of computer vision and natural language processing. It needs a computer to give a natural language answer based on pictures and questions asked about the pictures. So, the study looks at problems related to scams that have to do with network breaches. Current work by Siyu^57^ investigates how to improve the blending attention mechanism in visual question answering. Therefore, the study addresses network breaches and scam difficulties.

Ponzi scheme detection involves the collection of advertisements claiming high returns to participants on social media platforms and capturing their Ethereum addresses for analysis of their transactions for later identification^58^. Detecting Ponzi schemes on Ethereum is an urgent task, and a big challenge^22,59^. Previously, many works have been conducted on Ponzi scheme detection^60,61^. Some of the developed models have a problem with over-fitting, data imbalance, and prediction shift. The paper by Abiodun et al.^9^ employed feature selection optimization methods for optimal text classification to handle the problem of overfitting. This paper developed a detection model for Ponzi schemes using machine learning algorithms and feature engineering. Feature engineering was used to manipulate the data set in other to solve the problem of an imbalanced dataset, thereby improving the model’s training for better performance and greater accuracy. It uses a large dataset for the models’ training in order to handle prediction shift problems caused by target leakage. This model when deployed will be able to detect Ponzi schemes on time, therefore reducing investors’ risks in Nigeria.

## Methodology

In this section we outline the methodology and materials put together to achieve the goal of this research work, that is the research design, collection of the Ponzi scheme dataset, data cleaning/pre-processing, model training, and other tools used in this research work. Due to the openness of Ethereum, we were able to access the transaction history of Ponzi schemes. The method includes collecting a dataset from a popular data science site known as Kaggle.com. Thus, in this work, more than twenty thousand (20,000) dataset was collected from kaggle.com. The collected dataset includes information about each transaction such as the amount sent or received sender’s and receiver’s addresses, and the transaction time. Data cleaning and preprocessing were performed in other to handle the issue of inconsistent data and to improve the data efficiency for the Model’s training. More than 20,000 datasets were gathered for this work. Researchers can find all the code and data they need inside Kaggle to carry out data science work. So as to quickly complete any analysis, about over 50,000 accessible datasets, and 400,000 public notebooks are available on the Kaggle site to solve real-world ML problems. One of the weaknesses of the machine learning techniques (such as RF, KNN, and NN) used was that as the size of a neural network’s design grew, so did the amount of data required. But to overcome the weakness challenge, we chose not to reuse the data in order to achieve decent outcomes.

### Used classifiers

Due to fair comparison, three different classifiers, that is, RF, KNN, and NN for Ethereum fraud transactions with identical features were employed in this study.

#### Random forests

The Random Forests (RF) technique is a collaborative method of learning that may be used to perform classification or other tasks on a dataset by training and generating many trees based on the characteristics of the dataset. The class and probability formulas shown below are used to determine the Gini index of each branch on a node, which identifies the most likely branches.

#### K-Nearest neighbor

The K-Nearest Neighbor (KNN) clustering technique is commonly used in the data science sector to categorize datasets. The category for the datapoint is determined using the Euclidean distance algorithm. Consider the following scenario: We must assign the datapoint. This can be resolved using the KNN as follows:

- Randomly, select the K nearest neighbors.
- Using the Euclidean distance, find the nearest neighbor of the datapoint point that needs to be classified.
- Count how many data points are in each of these K groups.
- Assign the unclassified data point to the cluster of neighbors with the most neighbors.

#### Classifiers based on neural networks

A neural network (NN) is made up of units (neurons) that are organized in layers that convert an input vector into some output. Each unit receives an input, applies a (typically nonlinear) function to it, and then forwards the output to the next layer. In general, feed-forward networks are described as follows: a unit feeds its output to all units on the following tier, but there is no feedback to the preceding layer. Weightings are applied to signals as they flow from one unit to the next, and it is these weightings that are changed throughout the training phase to adapt a neural network to the specific task at hand. This is the stage of learning. Neural networks have found use in a wide range of situations. These range from function representation to pattern recognition, which is the topic of this article.

### Classification and detection/prediction

The proposed Ponzi scheme detection method was a machine learning classification which is a supervised learning technique. That is, the detection model was trained using a supervised learning approach, where the target is to classify each transaction as either a Ponzi scheme or not Ponzi. During the model training, three different machine learning algorithms were applied. The algorithms are KNN, RF, and NN algorithms, which are capable of handling both small and large datasets and complex relationships between the input features and target. After training three different models using the three selected machine learning algorithms, the models were evaluated for their performance using: accuracy, precision, recall, Kappa, and F1 score metrics to know the best model for Ponzi scheme detection. In our models’ training, all three models were trained using a tenfold cross-validation method. This means that the dataset was split into 10 consecutive folds—each fold was used as a validator once, while the remaining ninefolds served as the training set.

### Data cleaning/preprocessing

**Figure 2:**
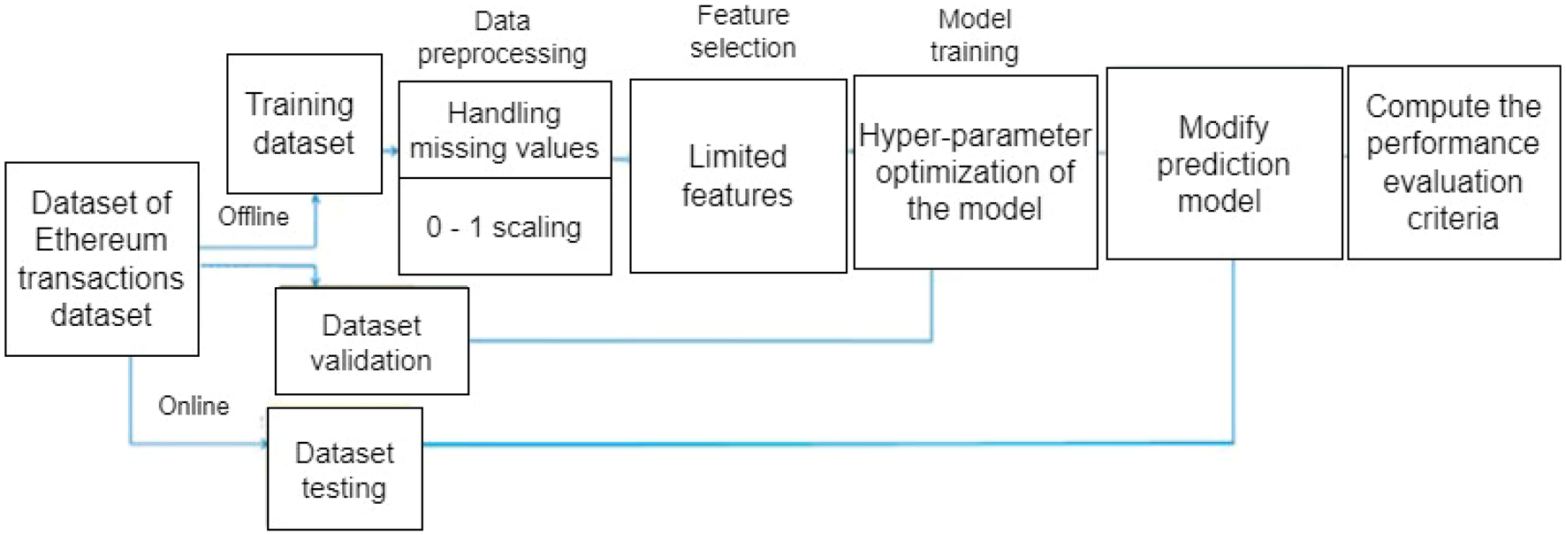
Proposed model workflow.

**Figure 3:**
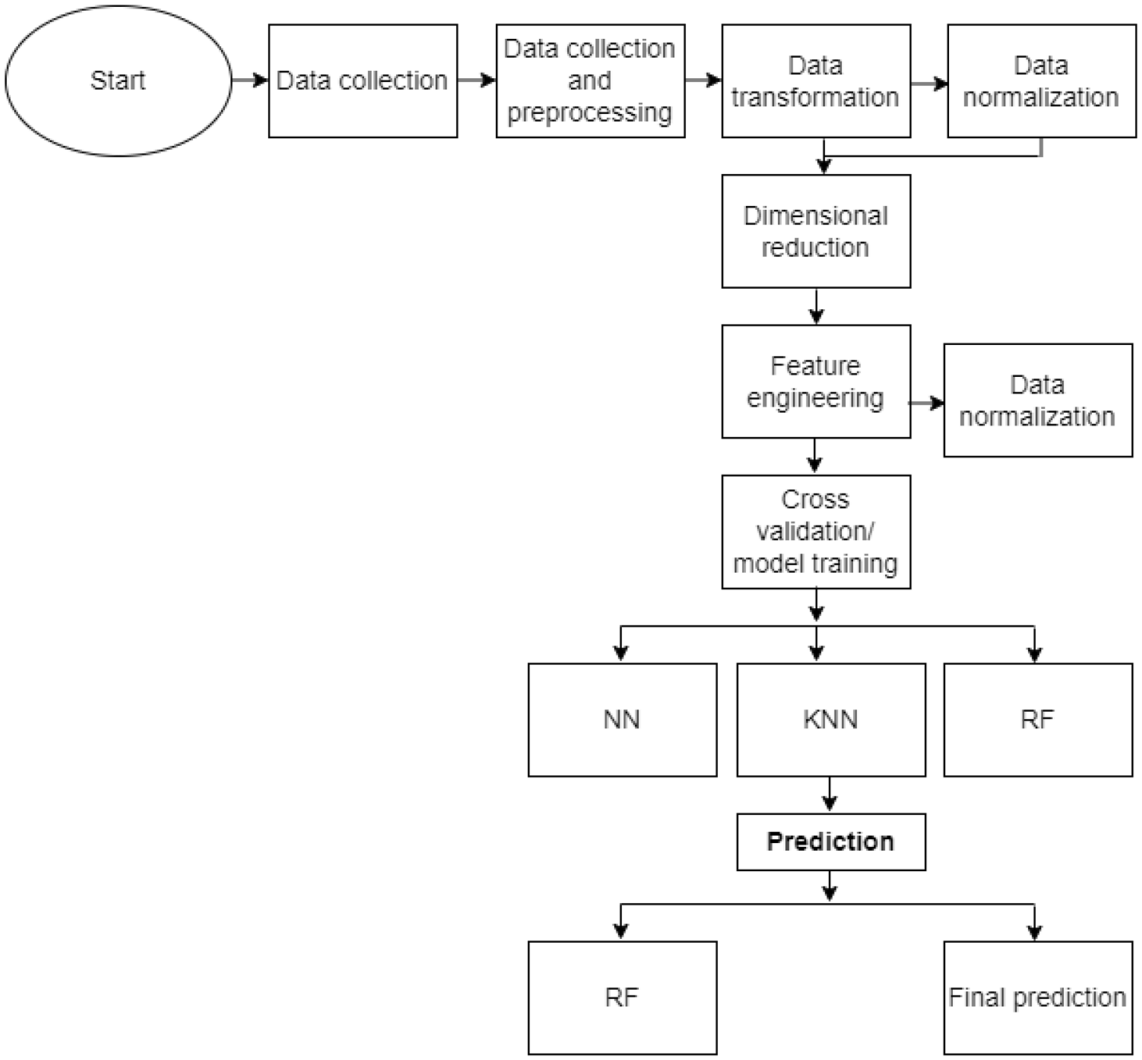
Data collection and analysis design flow of the machine learning models.

More than 20,000 datasets relating to Ethereum transaction was collected from Kaggle.com; it was preprocessed to make it suitable for our model’s training. This step was implemented in other to clean the data set in other to check for missing or inconsistent values and to remove any irrelevant or redundant information. The data was then normalized to ensure that it was in a suitable format for the machine learning algorithms to work with. Microsoft Excel was used to convert it to a comma-separated value (CSV) file. The CSV format was chosen for easy data pre-processing using Pandas after which the models were trained. Before training our models, the preprocessed data set was split into two: training and testing data sets respectively. The flowchart for the proposed model is presented in Fig. 2. Likewise, the network design flow diagram of the model is shown in Fig. 3.

### Feature selection

This section looks at how feature selection can be used to detect fraud in web transactions. The features were chosen based on their relevance to the problem at hand and their ability to accurately predict the likelihood of a transaction being Ponzi. The parameters of the model were then learned using a training set of data, which was used to fine-tune the model and improve its accuracy. We employed three feature selection strategies to do this analysis and evaluated how class imbalance can make this work challenging. In this stage, resampling strategies were used including a resampling method. In the feature selection step, we adopt a unique resampling technique as a possible solution to this problem. Five intelligent resampling algorithms were employed to detect anomalies before the feature selection process. A brief description of these strategies is provided in Table 1. Our anomaly-specific resampling algorithm is shown in pseudocode 1. Smote is used to delete uncommon negative class instances and replicate rare positive class instances. This method does not require class ratio information. The number of instances that meet conditions determines the ratio in the sampling outlier technique.

**Table 1:** Resampling methods adopted in the study.

S/n	Reference	Description	Method	Label	Type
1	This study resampling method was developed	A combination of under- and oversampling, using the pseudocode 1	Sample exclusion	Sampling outlier	Mixed
2	Chawla et al.^62^	Interpolates between pre-existing minority cases to create new artificial minority examples	Smote	Smote	Oversampling
3	Lima and Pereira^63^; Mani and Zhang^64^	Remove negatives with the least average distances to the three closest positive examples	NearMiss-1	Resampling method number 1 (RMN-1)	Under-sampling
4	Lima and Pereira^63^; Mani and Zhang^64^	Get rid of negative examples whose average distance from the three most distant positive examples is greater than three	NearMiss-2	Resampling method number 2 (RMN-2)	Under-sampling
5	Lima and Pereira^63^; Mani and Zhang^64^	Eliminate the bad examples to ensure that every good example has a few bad ones surrounding it	NearMiss-3	Resampling method number 3 (RMN-3)	Under-sampling

Logistic Regression^65^ and Bayesian Networks^66^ are state-of-the-art supervised classification methods we utilize to compare feature selection methods and uncover frauds. Decision Tree-J48 Implementation^67^. To compare models, our results are Sponzi scheme fraud detection via feature selection.

**Figure 4:**
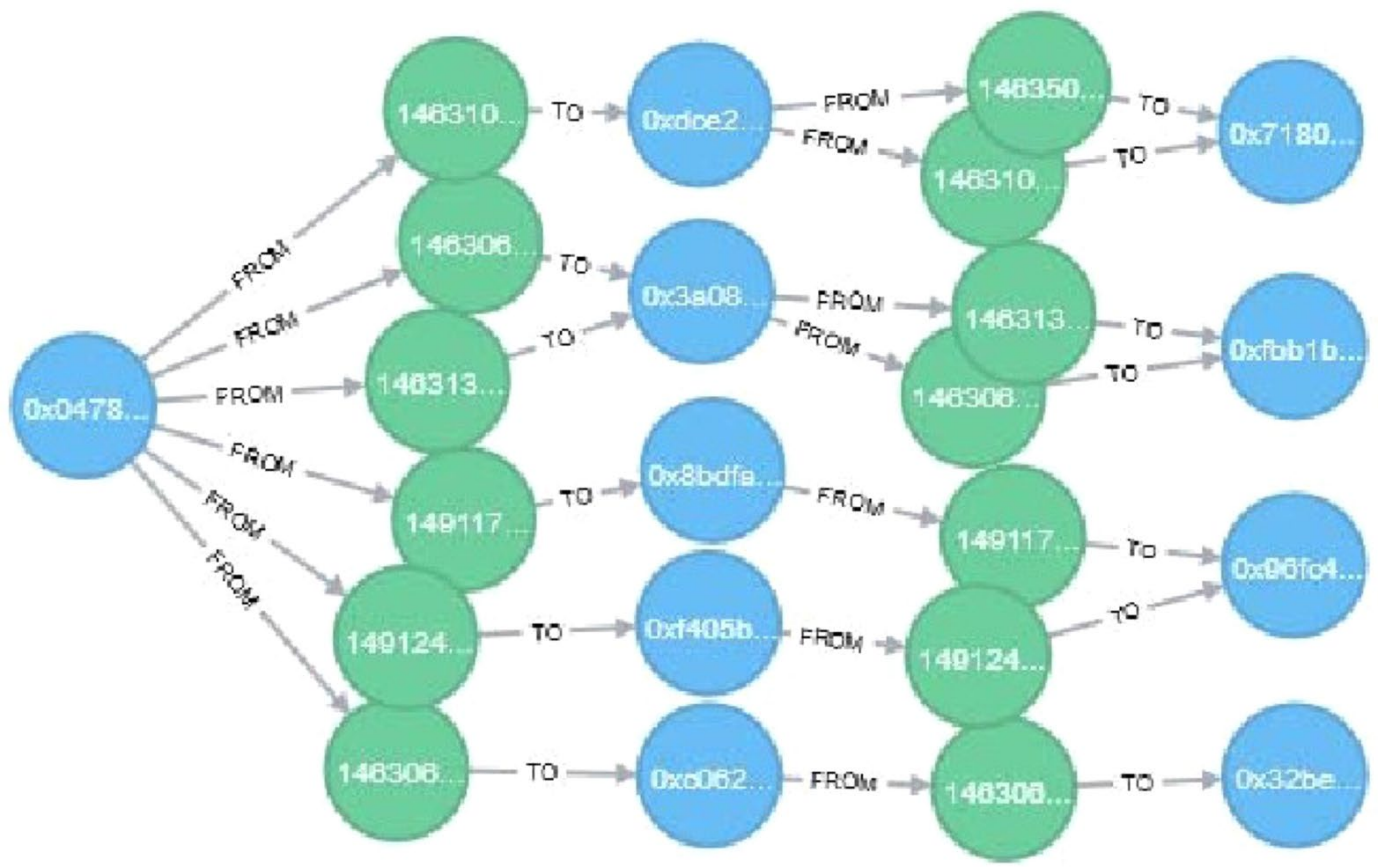
Ponzi scheme transaction graphs.

In this work, we equally selected some important features suitable for the model’s training. The accounts are shown in blue color, while transactions are shown in green color. The transaction graph can be represented as in Fig. 4.

### Metrics for evaluation

The four usual outputs of a Ponzi scheme contract detection algorithm are True Positive (TP), True Negative (TN), False Positive (FP), and False Negative (FN). TP stands for the number of correctly identified Ponzi scheme contracts, TN for correctly identified contracts without Ponzi schemes, FP for the number of smart contracts without Ponzi schemes that are incorrectly predicted as Ponzi scheme contracts, and FN for the number of Ponzi scheme contracts that are incorrectly predicted as contracts without Ponzi schemes. We measure the model’s performance using precision, recall, and F-score, which are introduced as follows:

1 $$Precision = \frac{TP}{{TP + FP}}$$

2 $$Recall = \frac{TP}{{TP + FN}}$$

3 $$F^-{\text{-}}score = 2 \times \frac{Precision \times recall}{{Precision + recall}}$$

The performance of the Ethereum fraud detection model was evaluated using a range of metrics, including accuracy, precision, recall, and F1-score. These metrics provide a clear and comprehensive view of the model’s performance and allow for comparisons with other models and algorithms. The use of these metrics ensures that the model is evaluated objectively, providing a clear understanding of its strengths and weaknesses. Another metric for evaluation was by extraction of features.

**Figure:**
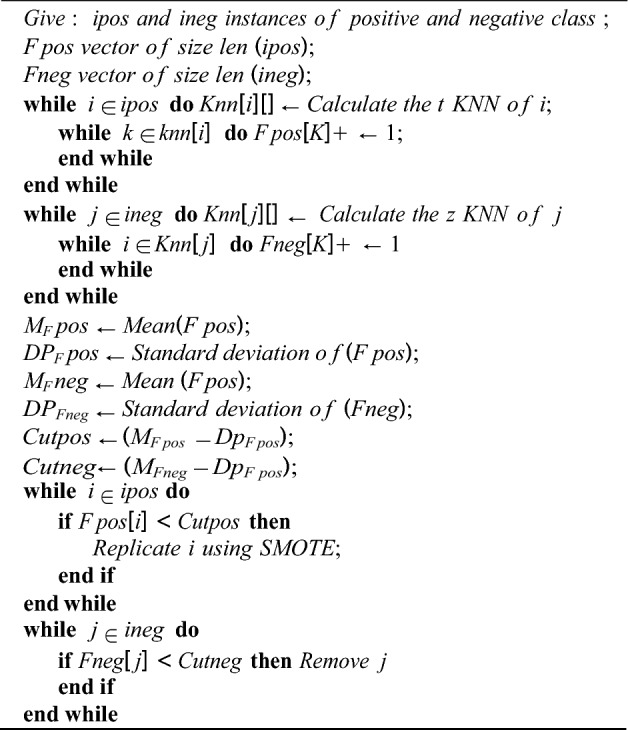
Algorithm 1 sampling outlier resampling method.

#### Area under the curve, average F1 and relative economic efficiency

We also analyzed the results using tree metrics:

1. The area under the curve (AUC) of false positive rate (FPR) and true positive rate (TPR) varies with the classification threshold.
2. Average F1 weights precision and recall. To compare results, we utilize the Average F-Measure (Avg F1) for each class (0 and 1).
3. Relative Economic Efficiency (Relative EE) calculates financial gains from fraud detection models using Eq. (4).
  4 $$EE = k,\;\;\;TNValue - \left( {\left( {1 - k} \right) \cdot FNValue + p \cdot FPValue} \right)$$
  where k is the company’s benefit for each transaction, p is the false positive penalty, and TNValue, FNValue, and FPValue are the total of true negative, false negative, and false positive transaction values.

#### Extraction of features

**Figure 5:**
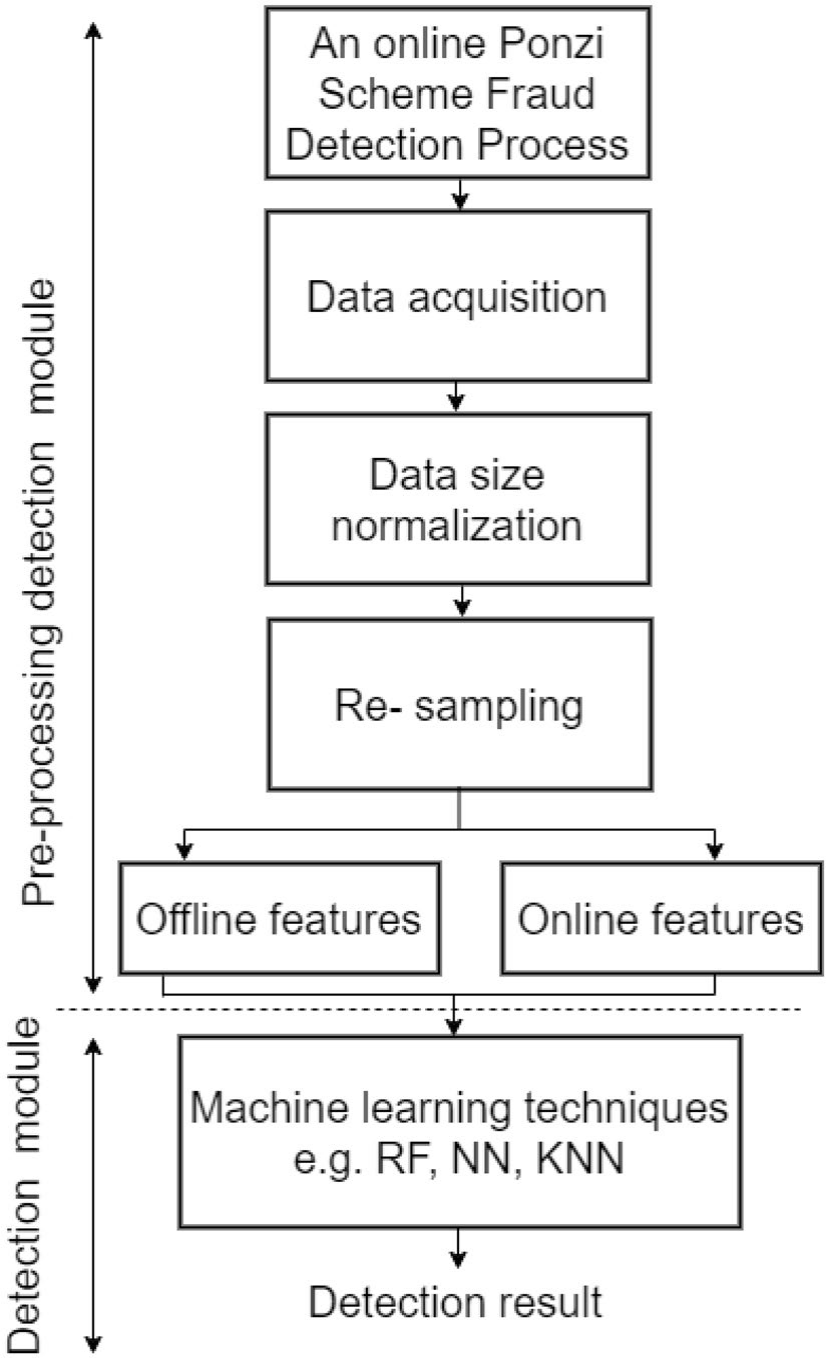
Feature extraction process.

The feature extraction process is represented in Fig. 5. Features were extracted from various views using the opcodes and contract developer information as inputs. The opcodes are a collection of tokenized mnemonic words, they may be thought of as documents that contain tokenized opcodes in the form of words. Thus, techniques for feature extraction in the context of natural language processing can be utilized for the opcode data. Therefore, statistical analysis is initially carried out before choosing which features to employ in order to discover which features could be used to develop an effective model.

First, using the Bag-of-Words model, term frequency count, and TF-IDF value are computed^68^. Two approaches see the documents as a collection of words that are not in any particular order. Also considered while calculating N-grams and TF-IDF for N-grams is the location of words in continuous sequences. This model’s term count feature offers a perspective of quantity information while retaining the document’s length as a potential source of information.

The N-gram feature is similarly derived by counting how many times all three N-grams appear in a document. However, as n increases, the number of N-gram sequences also increases exponentially. N-gram sequences with document frequencies of less than 0.1 are disregarded in order to limit the complexity of our model, where the document frequency stands for the reciprocal of IDF before taking the logarithm. The number of features before and after this selection procedure is shown in Table 2.

**Table 2:** Features before and after selection.

S/n	Class	Number of features (Before selection)	Number of features (After selection)
1.	Unigram	150	80
2.	Unigram + bigram	8850	767
3.	Unigram + bigram + trigram	13,532	1150

#### Statistical indicators

Some statistical indicators for measuring the performance of the ANN models were used in various research to evaluate the performance of ML algorithms such as RF, NN, and KNN. Take for instance, the usage of variance of absolute percentage error (VAPE), mean absolute percentage error (MAPE), mean absolute error (MAE), and root mean squared error (RMSE).

### Details of the data set

Ponzi schemes used on Ethereum are documented in a public dataset (Google/CvdxBp). The dataset is created by looking at the source code of contracts, which is consistent with our classification criteria. Beginning with the contracts whose source code is accessible on blockchain explorers, we locate 1000 Ponzi schemes among them. By searching the blockchain for contracts whose bytecode is strikingly similar to a contract that has already been labeled as a Ponzi scheme, we increase this collection to 1100 schemes. By personally reviewing their decompiled code, false negatives are eliminated. Most of the transactions originate from open-source smart contracts; hence we collected our dataset from these contracts. Also, we developed a Ponzi contract detector from the bytecode level. This concept was developed in light of the fact that Ethereum is home to a sizable number of latent Ponzi schemes as demonstrated by the studies conducted by^13,69^. A bytecode-level detector can be used to detect all smart contracts running on Ethereum. Hence, users can be warned before interacting with bytecode-only smart contracts or when adversaries try to deploy Ponzi contracts via e-wallets. Additionally, we discuss the potential validity risks posed by the biased distribution of the dataset when compared to the complete family of smart contracts on the Ethereum Dataset.

## Experimental result

Here in this section, we present our experimental results. Which includes experiment settings, importance feature selection, and evaluation metrics.

### Result of the dataset

Main information about this dataset Table 3.

**Table 3:** Main information about the dataset.

Dataset	Result
Located Ponzi schemes	1000
Increased collection of schemes	1150
Number of features	80
Number of continuous features	767
Number of categorical features	1100
Ponzi scheme fraud proportion	0.8
Period of analysis	2021/2022

### Feature performance

The detection of Smart Ponzi schemes using the three types of characteristics. A comparison of feature performance is presented in Table 4. Table 4 allows for several deductions, first and foremost, the account surprisingly, characteristics are ineffective at detecting clever Ponzi schemes. We anticipated that the account features would be useful performance since intelligent Ponzi schemes operate differently. However, the low recall demonstrates that the model based on these features is nearly useless. In comparison, the opcode features are as expected quite efficient. One possible explanation for this outcome is that many smart contracts are experimental, making it difficult to discern their kind from behavior. Many smart contracts have no transactions. Another possible explanation is that the amount of account characteristics is insufficient. Second, the relevant measurements demonstrate that modes based just on opcode properties can be utilized to detect smart Ponzi schemes. Finally, the model’s performance can be increased by integrating opcode and account features.

**Table 4:** A comparison of feature performance based on three measurable parameters.

S/n. Features	F-score	Recall	Precision
1. Account	0.44	0.32	0.74
2. Opcode	0.84	0.80	0.90
3. Account + Opcode	0.86	0.81	0.94

### Feature selection

**Figure 6:**
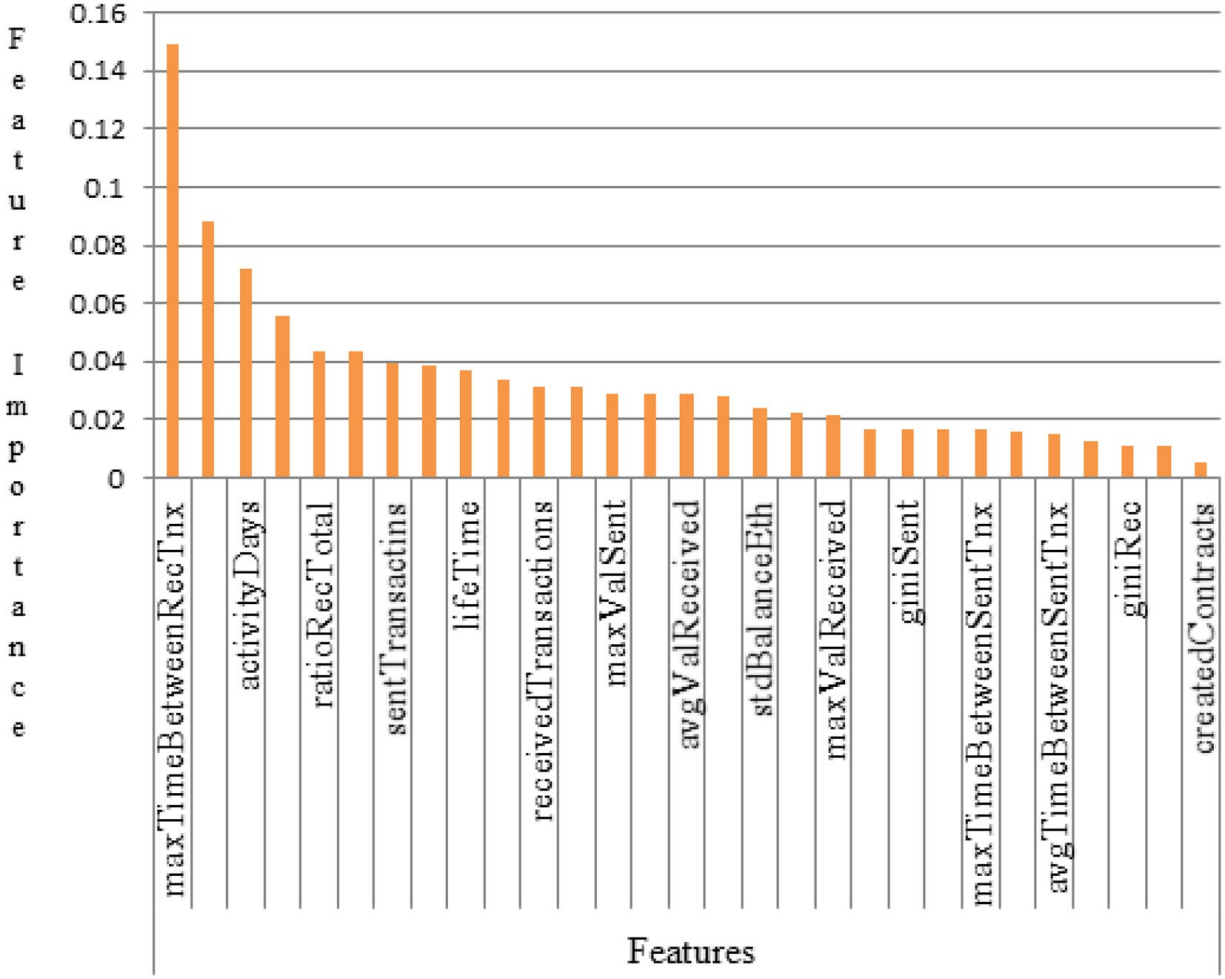
Extracted selected features based on their importance in the model’s training.

In our experiment, extracted features were selected based on their importance in the model’s training and plotted the graph as shown in Fig. 6. It is clear that ‘maxTimeBetweenRecTnx’, ‘avgValSent’, and ‘activityDays’ have a huge impact on the final decision. MaxTimeBetweenRecTnx (maximum time between received transaction). This means the total time taken to receive any transaction made. This is the most important feature because, the faster the transaction, the larger the participants at a given time. AvgValSent (average value sent). This means the average of the amount sent to participants within a given period. activity Days. These are the number of days the Ponzi scheme operated before its collapse.

### Performance of the three models trained

**Figure 7:**
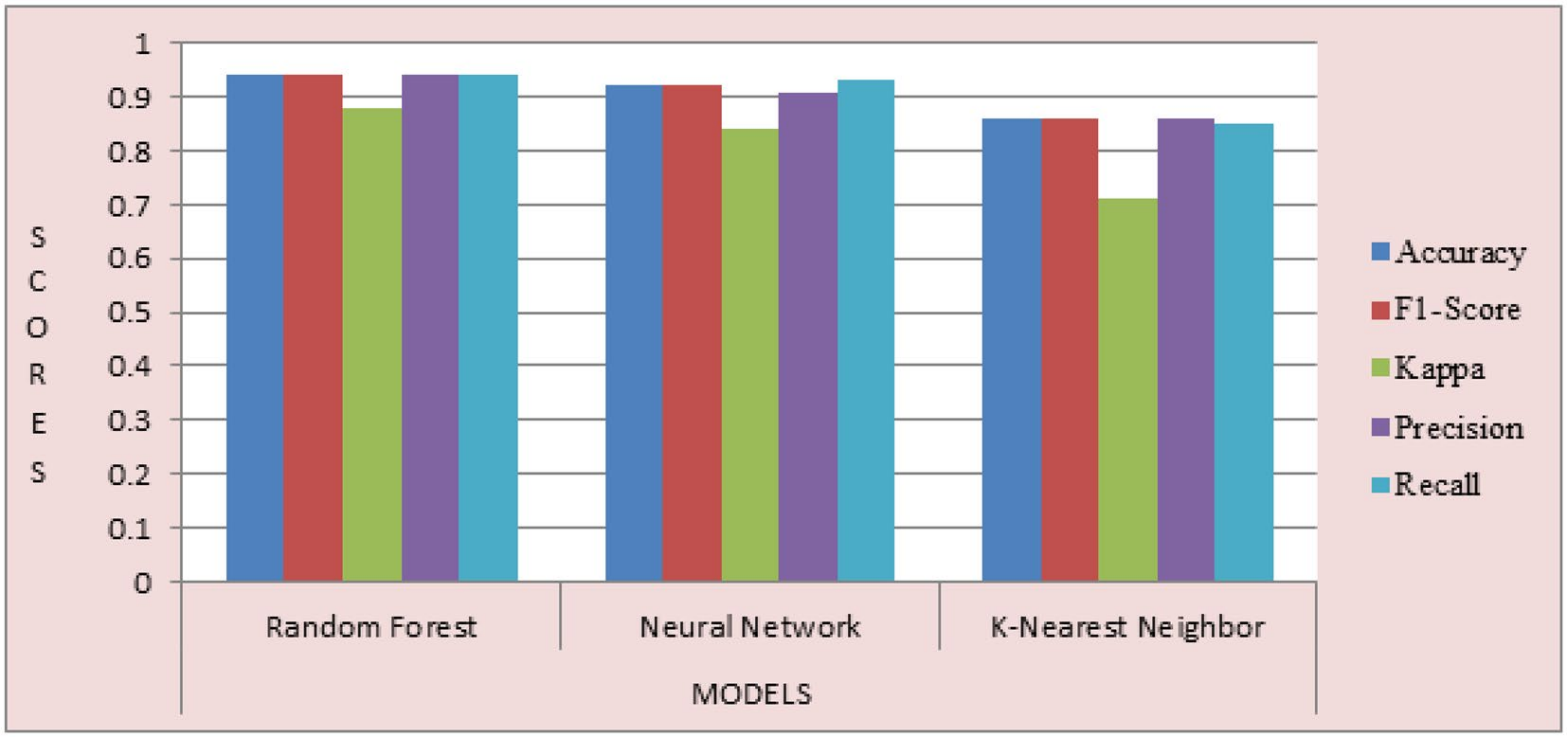
The result of the three compared models.

**Figure 8:**
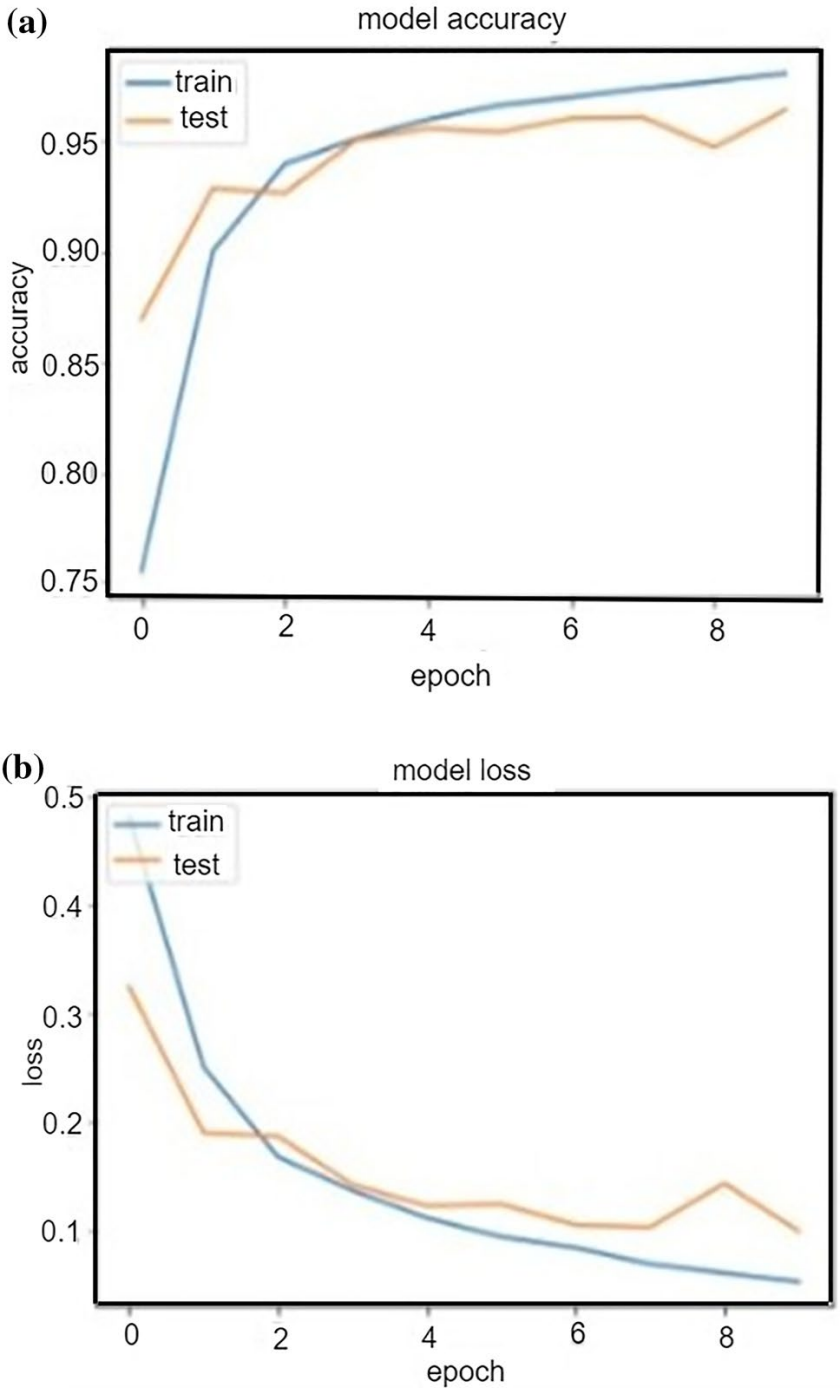
Accuracy and loss found in the model.

The result of the proposed classifiers was evaluated and presented. Performance evaluation was used to ascertain how the three models work. To achieve this, we applied the following metrics: Accuracy, Recall, F1-score, Kappa, and Precision. The results are shown in Table 5. Likewise, the graph showing the result of the three compared models can be highlighted in Fig. 7. It is clear from Fig. 7 that the RF model outperforms the other two models, followed by NN, while KNN has the lowest scores. Furthermore, the graph showing the model accuracy and loss is shown in Fig. 8 (a) and (b).

**Table 5:** Comparison of the performance of the three models trained.

Models	Accuracy	F1-score	Kappa	Precision	Recall
RF	0.94	0.94	0.88	0.94	0.94
NN	0.92	0.92	0.84	0.91	0.93
KNN	0.86	0.86	0.71	0.86	0.85

### Other classifiers with our proposed model

**Figure 9:**
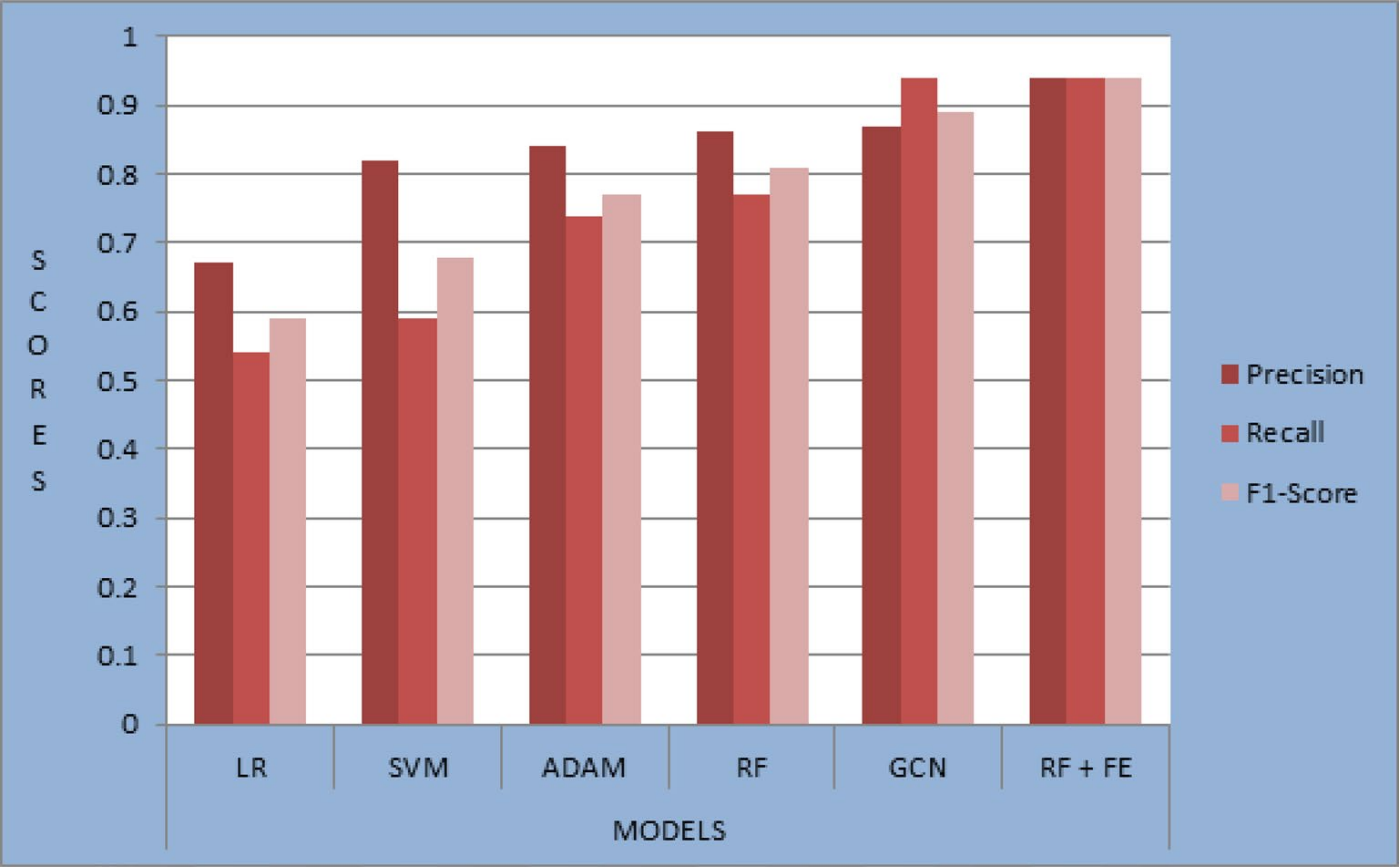
Representation of the previous model with our proposed model.

Table 6 presents the evaluation and results of the trained machine learning models, including random forest, support vector machine, logistic regression, ADAM, and GCN. These classifiers were assessed and compared to determine their performance in detecting Ponzi schemes on the Ethereum platform. Figure 9 shows that the proposed model (RF + FE) outperforms other models with a precision of 0.94, recall of 0.94, and F1-score of 0.94 respectively.

**Table 6:** Result of other classifiers with our proposed model.

Classifier	Precision	Recall	F1-Score
Logistic regression	0.67	0.54	0.59
Support vector machine	0.82	0.59	0.68
ADAM	0.84	0.74	0.77
Random forest	0.86	0.77	0.81
GCN	0.87	0.94	0.89
RF + feature engineering	0.94	0.94	0.94

Moreover, the representation of the previous model with our proposed model can be compared as shown in Fig. 9.

### The confusion matrix of our proposed model

**Figure 10:**
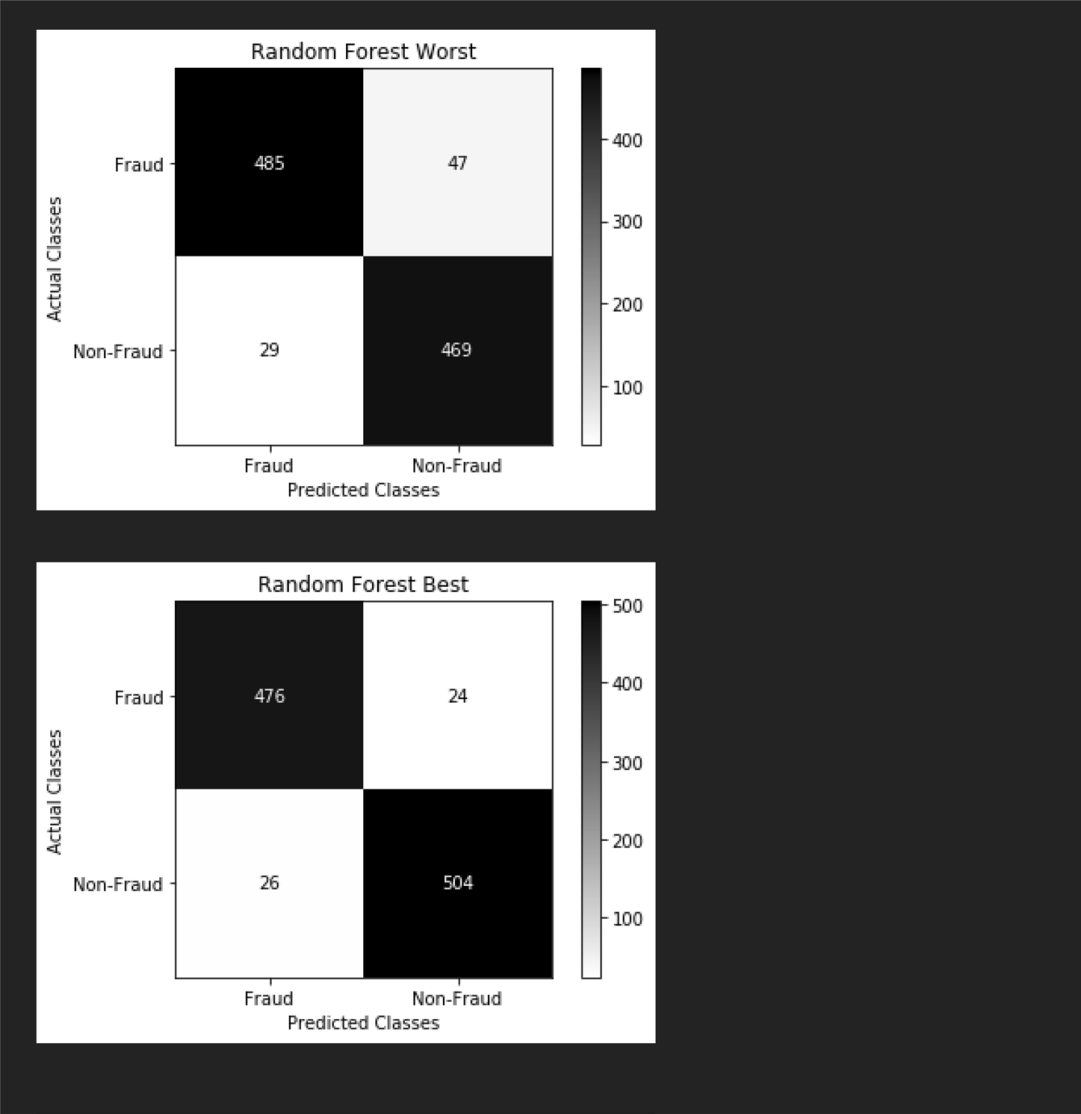
The confusion matrix of our proposed model.

The confusion matrix (CM) is a layout that helps visualize the multiple outcomes of a classification problem’s detection and findings. The graph of all the predicted and actual values of a classifier is presented in Fig. 10. The accuracy indicates how "right" our forecasts are. In this scenario, the model predicted correctly 94% of the time.

### Comparison of machine learning algorithms and the baselines

The developed machine learning algorithms were compared with the conventional feature-based Ponzi scheme contract detection techniques, including Account, Opcode, and Account + Opcode, to demonstrate the superiority of machine learning techniques for contract detection. The results of precision, recall, and F-score values are presented in Table 7. The table presented the success rate of various Ponzi scheme contract detection techniques. We can see from the result on the above graph that the RF model outperforms the other two models, followed by NN, while KNN has the least performance.

**Table 7:** The success rate of various Ponzi scheme contract detection techniques.

Method	Precision	Recall	F-Score
Account	0.54	0.30	0.40
Opcode	0.84	0.75	0.82
Account + Opcode	0.92	0.69	0.79
Machine learning algorithms	0.98	0.87	0.90
RF	0.94	0.94	0.94
NN	0.91	0.93	0.92
KNN	0.86	0.85	0.86

### Proposed model compared with previous models

**Figure 11:**
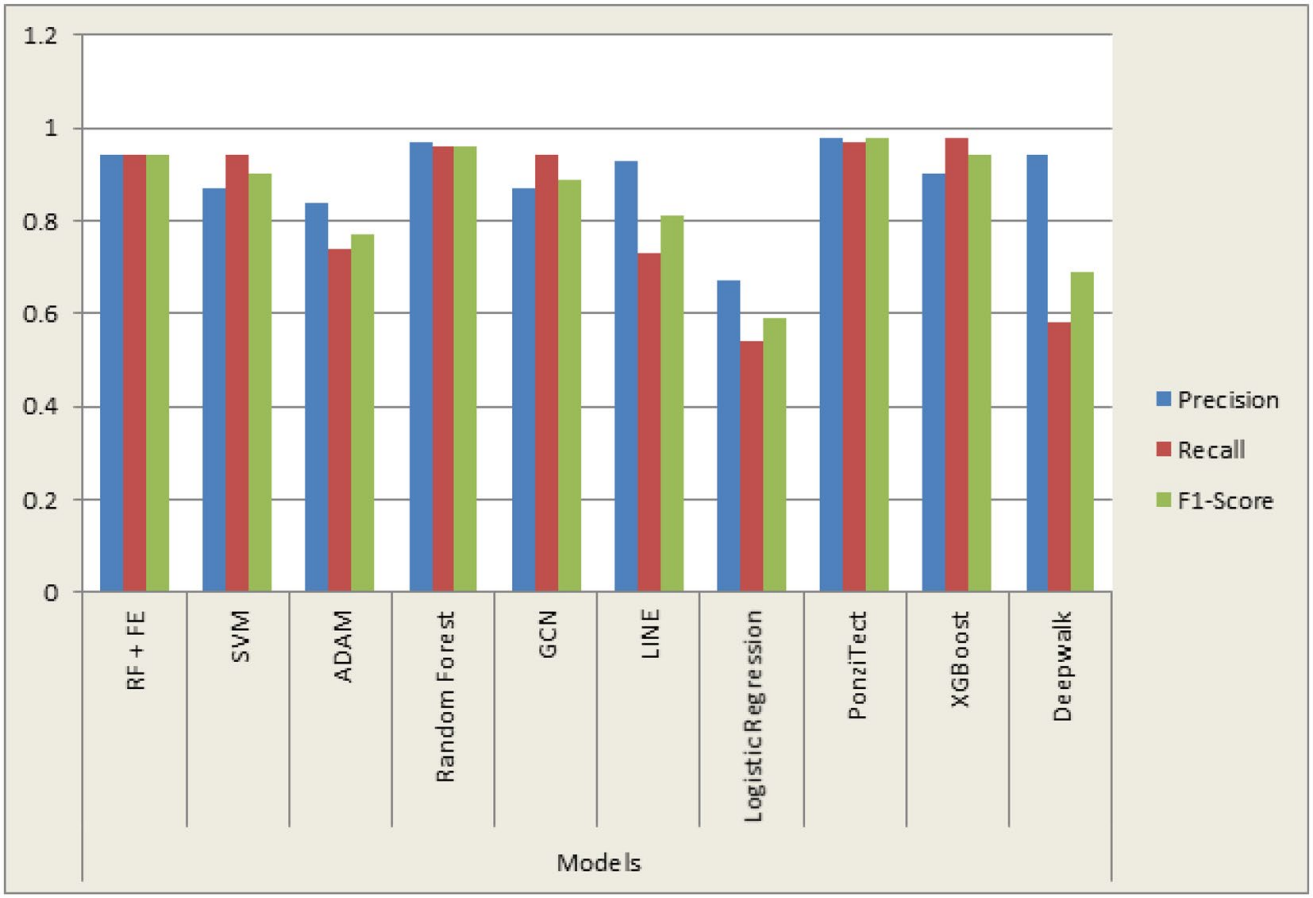
Proposed model compared with previous models.

In addition, the results of different models trained by previous researchers^40,60^ were compared as shown in Table 8. Therefore, from the result, this work’s proposed model RF is the third in the range in terms of Precision, F-score, and Recall, with 94% accuracy. Likewise, the proposed model compared with previous models is shown in Fig. 11.

**Table 8:** Result of other classifiers with our proposed ML model.

Methods	Precision	Recall	F1-Score
RF + feature engineering	0.94	0.94	0.94
Support vector machine	0.87	0.94	0.90
ADAM	0.84	0.74	0.77
Random forest	0.97	0.96	0.96
GCN	0.87	0.94	0.89
LINE	0.93	0.73	0.81
Logistic regression	0.67	0.54	0.59
PonziTect	0.98	0.97	0.98
XGBoost	0.90	0.98	0.94
Deepwalk	0.94	0.58	0.69

## Discussion of the result

The decentralized nature of Ethereum has made it looks like a reliable avenue for Ponzi scheme scammers and other fraudsters to breed in. However, due to the openness of Ethereum, researchers can trace their fraudulent activities on Ethereum. Ethereum is ranked as the second most used cryptocurrency after Bitcoin.

1. In-depth research has been done on malware detection methods for conventional software systems. Decentralized apps are a new class of software systems that have arisen because of the development of blockchain technology. A form of application made up of smart contracts that use the Ponzi scheme logic (known as smart Ponzi schemes) has caused irreparable loss and slowed the advancement of blockchain technology. Although they typically had a brief lifespan, these smart contracts involved sizable sums of money. While it has been crucial to detect these Ponzi schemes before they cause financial harm, current methods suffer from three main flaws: an inadequate dataset, a reliance on transaction records, and a low level of accuracy.
  Thus, in this study we first created a larger dataset, then, a significant number of features are extracted from various views, including bytecode, semantics, and developers. The transaction records have no bearing on these features. In addition, we used machine learning techniques to construct our identification model. The proposed method’s ability to detect clever Ponzi schemes from their inception is its main strength. Then, a significant number of features are extracted from various views, including semantic, bytecode, and developers. The transaction records have no bearing on these features. In addition, we used machine learning techniques to construct our identification model. The proposed method’s ability to detect clever Ponzi schemes from their inception is its main strength.
2. The best performance across all evaluation metrics is shown by the machine learning algorithms, demonstrating the supremacy of performance. Take for example, it earns an F-score of 0.90, a precision value of 0.98, and a recall value of 0.87. The Machine learning algorithms perform better than Account in terms of precision, recall, and F-score by 98%, 87%, and 90%, respectively. While Accounting in terms of precision, recall, and F-score by 54%, 30%, and 40%, respectively. In terms of precision, recall, and F-score, the Machine learning algorithms outperform Opcode by 84%, 75%, and 82%, respectively. Additionally, in terms of precision, recall, and F-score, the machine learning algorithms perform better than Account + Opcode by 92%, 69%, and 79%, respectively. In conclusion, the results of the trial demonstrate the Machine learning algorithm’s strong capacity to automatically learn structural and semantic elements from the smart code.
3. The random forest model performs the worst across all assessment criteria when it is trained using only the Account features. The Ponzi scheme contract detection algorithm based on the Account characteristics is almost useless given the poor recall value (0.20). The performance of the random forest model is high when the Opcode features are used in the training process. The accuracy of the model is further increased but the recall and F-score values decrease when the Account features and Opcode features are used to train the random forest model. However, in comparison of the three cutting-edge models of the machine learning algorithms in Table 1, that is, random forest, neural network, and k-nearest neighbor. The random forest achieves the highest Accuracy, F1-score, Kappa, Precision, and Recall values of 94%, 94%, 88%, 94%, and 94% respectively.
  Selecting an algorithm for training a Ponzi scheme detection model is an important factor that needs to be considered to get a good performance. Based on this, the researcher considered a wide range of algorithms such as Random Forest (RF), Neural Network (NN), and K-Nearest Neighbor (K-NN). After the model’s training, the obtained results were compared as shown in Table 1. The result in Table 1 and Table 3 is that Random Forest (RF) has the best performance among the selected algorithms with accuracy, F-score, Precision, and Recall of 94%. It revealed an association between the inflow and outflow of contracts over time. Finally, we gauge the number of transactions per month. Hence it is more suitable for Ponzi scheme detection, and thus we choose it as an algorithm in this research.

There are a few lessons from the Ponzi scheme which include;

1. The Ponzi scam brings in new investors by promising them a substantial payoff with little to no risk, which creates returns for previous investors.
2. The fraudulent investment scheme’s basic idea is to reimburse the initial backers with money from future investors.
3. Companies that run Ponzi schemes concentrate all of their efforts on finding new investors because, without them, the scheme will run out of money.
4. The Securities and Exchange Commission (SEC) has provided advice on potential Ponzi scheme red flags, such as guarantees of returns or unregistered investment vehicles with the SEC.

## Conclusion

The study creates a classification model to identify hidden Ponzi schemes implemented as smart contracts by first extracting features from user accounts and operation codes of the smart contracts and then confirming them on Ethereum. The experimental results demonstrate the great precision that the suggested approach may attain for practical use. What’s more, the method can be used to spot Ponzi schemes even as they’re being created. We calculate that there are more than 400 Ponzi schemes active on Ethereum using the suggested method. Based on these findings, we suggest developing a standard platform to assess and keep track of each smart contract that is formed to spot fraud in its earliest stages.

The aim of this research was to train an effective Ponzi scheme detection model that can detect Ponzi schemes on time so that the public will be warned against the danger of investing in such schemes. To achieve this, three different models were trained using machine learning algorithms (RF, NN, and KNN) and feature engineering after which their performances were evaluated to ascertain the most effective model using performance metrics. Based on their performances, the proposed model is the RF model which outperforms the two other models in terms of Accuracy, Precision, Kappa, and F1-score.

The proposed RF model was also compared to previously developed models, and our proposed model has shown a slight difference in terms of Precision and F1-score. From the results and findings, it can be deduced that the research objectives were able to treat the research problems. Our Ponzi scheme detection model can detect Ponzi schemes at their early stage. Our model was able to tackle most of the problems encountered by previous models, such as: over-fitting in which we applied careful feature selection in other to solve, we used data augmentation and normalization to solve the problem of data imbalance and to handle the problem of prediction shift, and we employed large dataset for our models’ training. In addition to successfully reducing the dataset of Ponzi schemes’ 70 features to just 10, this innovative study also maintained a very high level of accuracy. The key advantage of the suggested method is that it can effectively detect Ponzi schemes from the beginning.

Future work may consider the Bitcoin blockchain as this work only considers the Ethereum transaction network. It may also be of interest that the data set can be downloaded from another source apart from kaggle.com to train machine learning algorithms for another set of models to be developed.
