# Author Correction: Detection of Ponzi scheme on Ethereum using machine learning algorithms

**DOI:** 10.1038/s41598-023-48310-2

**Published:** 2023-11-28

**Authors:** Ifeyinwa Jacinta Onu, Abiodun Esther Omolara, Moatsum Alawida, Oludare Isaac Abiodun, Abdulatif Alabdultif

**Affiliations:** 1 Department of Computer Science, University of Abuja, Gwagwalada, Nigeria; 2 Department of Computer Sciences, Abu Dhabi University, 59911 Abu Dhabi, United Arab Emirates; 3 Department of Computer Science, College of Computer, Qassim University, 52571 Buraydah, Saudi Arabia

Correction to: *Scientific Reports* https://doi.org/10.1038/s41598-023-45275-0, Published online 27 October 2023

The original version of this Article contained an error in the name of the author Ifeyinwa Jacinta Onu, which was incorrectly given as Onu Ifeyinwa Jacinta.

The original Article has been corrected.

